# Design of novel graded bone scaffolds based on triply periodic minimal surfaces with multi-functional pores

**DOI:** 10.3389/fbioe.2025.1503582

**Published:** 2025-02-12

**Authors:** Rongwu Lai, Jian Jiang, Yi Huo, Hao Wang, Sergei Bosiakov, Yongtao Lyu, Lei Li

**Affiliations:** ^1^ Department of Spinal Surgery, Central Hospital of Dalian University of Technology, Dalian, China; ^2^ School of Mechanics and Aerospace Engineering, Dalian University of Technology, Dalian, China; ^3^ Department of Orthopaedic Surgery, Shengjing Hospital of China Medical University, Shenyang, Liaoning, China; ^4^ Faculty of Mechanics and Mathematics, Belarusian State University, Minsk, Belarus; ^5^ DUT-BSU Joint Institute, Dalian University of Technology, Dalian, China

**Keywords:** bone scaffold, triply periodic minimal surface, multi-functional pore, mechanical behavior, mass transport capacity

## Abstract

**Background:**

Various mechanical and biological requirements on bone scaffolds were proposed due to the clinical demands of human bone implants, which remains a challenge when designing appropriate bone scaffolds.

**Methods:**

In this study, novel bone scaffolds were developed by introducing graded multi-functional pores onto Triply Periodic Minimal Surface (TPMS) structures through topology optimization of unit cell. The performance of these scaffolds was evaluated using finite element (FE) analysis and computational fluid dynamics (CFD) method.

**Results:**

The results from FE analysis indicated that the novel scaffold exhibited a lower elastic modulus, potentially mitigating the issue of stress shielding. Additionally, the results from CFD demonstrated that the mass transport capacity of the novel scaffold was significantly improved compared to conventional TPMS scaffolds.

**Conclusion:**

In summary, the novel TPMS scaffolds with graded multi-functional pores presented in this paper exhibited enhanced mechanical properties and mass transport capacity, making them ideal candidates for bone repair. A new design framework was provided for the development of high-performance bone scaffolds.

## 1 Introduction

The high prevalence of orthopedic diseases worldwide underscores the urgent clinical need for bone scaffolds that demonstrate excellent performance (Campana et al., 2014; Wallace et al., 2017; Li Y et al., 2018). Currently, there is a demand for functionally graded bone scaffolds to facilitate the transition from cancellous bone to cortical bone in the gradient region of human bone defects (Fernandez de Grado et al., 2018). Nowadays, triply periodic minimal surface (TPMS) bone scaffolds are widely developed in bone implants (Davoodi et al., 2020). However, scaffolds designed based on TPMS structures cannot fully address two critical issues, i.e., the stress-shielding effect caused by a high elastic modulus and the insufficient mass transport capacity due to low permeability (Jiang et al., 2024; Vijayavenkataraman et al., 2020).

Since the performance of TPMS bone scaffolds mainly depends on their geometrical structure, how to improve the performance of bone scaffolds through rational structural design has become the focus and difficulty in current research studies. The high elastic modulus of bone scaffolds can cause the implanted artificial bone scaffolds to bear most of the mechanical loads, which resulted in a stress-shielding effect and further led to interface loosening (Barba et al., 2019). For functionally graded bone scaffolds, selecting an appropriate gradient transition is critical for achieving optimal performance, which remains a key challenge in current research studies. Peng et al. (2023) highlighted that the elastic modulus and permeability of bone scaffolds represent conflicting performance requirements, making it difficult to simultaneously optimize both properties. Moreover, seldom has research been conducted to completely address this problem. Therefore, it is a primary focus to resolve this trade-off issue in current studies. Although the elastic modulus of TPMS bone scaffolds is lower than that of traditional cubic porous structures, it is still higher than that of cancellous bone. Sevilla et al. (2007) reported that the elastic modulus of cancellous bone was 1.08 GPa. Wu et al. (2018) investigated the elastic modulus of cancellous bone under different loading directions. The results indicated that the modulus of cancellous bone was 3.47 GPa in the longitudinal direction and 2.57 ± 0.28 GPa in the transverse direction. Although there were discrepancies in the elastic moduli of cancellous bone between the studies, it was generally agreed that the elastic modulus of a bone scaffold should not exceed 3.00 GPa to align with that of the cancellous bone. Khaleghi et al. (2021) reported that the elastic modulus of a Schwarz P (i.e., one type of TPMS) scaffold with a porosity of 70% was 5.60 GPa, which was greater than that of cancellous bone. Rabiatul et al. (2021) stated that the permeability of cancellous bone is in the range of $3.66\times 10^{-8}m^{2}$ to $1.90\times 10^{-7}m^{2}$ . However, Santos et al. (2020) found that the permeability of a TPMS structure is in the range of $4.31\times 10^{-10}m^{2}$ to $8.44\times 10^{-9}m^{2}$ . Accordingly, it is necessary to optimize the topologies of TPMS bone scaffolds.

Recent studies on functionally graded TPMS bone scaffolds have made significant progress in improving gradient transitions to enhance both mechanical and biological performances. Wang et al. (2022) demonstrated that scaffolds with graded porosities improved load-bearing capacity while maintaining a high permeability, but it cannot be fully ensured that the challenges in optimizing the gradient transition are mitigated to prevent stress shielding. Zhang et al. (2020) found that mechanical strength and fluid transport were balanced by continuous gradient designs but struggled with achieving excellent performance by different porosities. Kim et al. (2020) reported that cell proliferation was enhanced using graded TPMS scaffolds. However, the mass transport capacity was limited by the low permeability. Li et al. (2021) improved mechanical anisotropy and acknowledged that achieving an ideal balance between elastic modulus and permeability for bone scaffolds remains a key challenge. Xu et al. (2020) highlighted that although graded designs show potential in addressing the mismatch of elastic modulus, further improvement was also needed to ensure mechanical stability and clinical applicability. The limitations of achieving an optimal balance between mechanical properties and permeability still exist, and therefore improved design strategies are needed.

In this study, a novel graded bone scaffold with multi-functional pores was developed based on the Schwarz P structure. The finite element (FE) analysis and computational fluid dynamics (CFD) method were applied to evaluate the performance of the novel functionally graded bone scaffolds with different porosities, and it was found that its mechanical properties and mass transport capacities were more excellent than those of conventional scaffolds, which indicated that novel bone scaffold may be a better candidate for bone repair (Hollister and Kikuchi, 1994).

## 2 Methodology

In this section, the Schwarz P structure is introduced, followed by a detailed description of the methodology for designing novel TPMS bone scaffolds with multi-functional pores. Methods for evaluating the mechanical properties of TPMS bone scaffolds were then described, including the evaluations of the elastic modulus and the anisotropy of the novel scaffolds using numerical homogenization methods. Finally, a numerical simulation method to evaluate the permeability is presented.

### 2.1 Design of novel TPMS bone scaffolds with graded multi-functional pores

A minimal surface has the smallest area subject to certain constraints and is mathematically defined as a surface with a mean curvature of 0 at any point (Yoo, 2011). TPMS is a minimal surface that possesses periodical array in three orthogonal base directions, and its topology is determined by functional expressions. Common TPMS structures include Schwarz P, Gyroid, Diamond, and I-WP (Blanquer et al., 2017). Schwarz P was proposed by the scientist Schwarz who first introduced the concept of minimal surfaces in 1883 (Strömberg, 2021). The continuous surface structure of TMPS reduces the stress concentration, and thus the stress bearing is more uniform. Meanwhile, the continuous surface structure of TPMS has better connectivity and larger specific surface area, and it was shown by cell culture that bone marrow stromal cells (BMSCs) exhibited better adhesion, proliferation, and osteogenic differentiation behaviors on the TPMS structural scaffold (Guo et al., 2023). Among the various TPMS structures (Diamond, Gyroid, Fischer–Koch S, etc.), the Schwarz P possesses cubic symmetry (Lu et al., 2019; Lu et al., 2020) and is more suitable for opening multi-functional pores in its surfaces, which is one major novelty in the present study. Therefore, the Schwarz P structure was selected as the example for structural design in this study. The Schwarz P structure was created by incorporating the thickness of the minimal surface. The Schwarz P structure can be characterized by the following mathematical function (Blanquer et al., 2017).

$$f\left(x,y,x\right)=\cos\left(\frac{2\pi }{n}x\right)+\cos\left(\frac{2\pi }{n}y\right)+\cos\left(\frac{2\pi }{n}z\right)-c,$$ (1)

where f determines the TPMS topology type; x,y,z are the coordinates of a point in the design space; n denotes the length of a unit cell; and constant c is used to control the two-phase domain, which determines the porosity of the structure (Peng et al., 2023).

The mass transport capacity of bone scaffolds depends on the pore size and the obstructed area (Kurtz et al., 2007; Ali et al., 2020). The structures with different porosities can be formed using various parameters, as illustrated in Equation 1. However, parameter c in Equation 1 is a unique variable to control the surface of the unit cell, which is related to porosity. Consequently, it was noted that multi-functional pores have been integrated into the scaffolds to enhance the mass transport capability and reduce the elastic modulus. Next, the process of generating multi-functional pores by the structural optimization method was briefly analyzed, and the differences between the gradient transition methods commonly used in previous studies for bone scaffolds and the newly proposed multi-functional pore gradient transition methods were described in detail.

The constitutive relation of stress σ and strain ε of the Schwarz P structure can be expressed as Equation 2. Since Schwarz P has cubic symmetry with three independent elastic constants, the stiffness matrix can be simplified as follows (Gere and Goodno, 2009).

$$\left(\begin{array}{c} \sigma_{11}\\ \sigma_{22}\\ \sigma_{33}\\ \sigma_{12}\\ \sigma_{13}\\ \sigma_{23} \end{array}\right)=\left[\begin{array}{cccccc} C_{11} & C_{12} & C_{12} & 0 & 0 & 0\\ C_{12} & C_{11} & C_{12} & 0 & 0 & 0\\ C_{12} & C_{12} & C_{11} & 0 & 0 & 0\\ 0 & 0 & 0 & C_{44} & 0 & 0\\ 0 & 0 & 0 & 0 & C_{44} & 0\\ 0 & 0 & 0 & 0 & 0 & C_{44} \end{array}\right]\left(\begin{array}{c} \varepsilon_{11}\\ \varepsilon_{22}\\ \varepsilon_{33}\\ \varepsilon_{12}\\ \varepsilon_{13}\\ \varepsilon_{23} \end{array}\right),$$ (2)

where $C_{11},C_{12}$ , and $C_{44}$ are the three independent elastic constants of the Schwarz P structure.

The derived expression for the elastic modulus of the Schwarz P structure is given by a computational simplification with the introduction of boundary conditions as Equation 3 (Ma et al., 2021; Feng et al., 2021; Lee et al., 2017).

$$E=\frac{18w^{\left(1\right)}w^{\left(2\right)}-2{\left[w^{\left(2\right)}\right]}^{2}}{3V\left[3w^{\left(1\right)}+w^{\left(2\right)}\right]}=\frac{18v_{\varepsilon }^{\left(1\right)}v_{\varepsilon }^{\left(2\right)}-2{\left[v_{\varepsilon }^{\left(2\right)}\right]}^{2}}{3\left[3v_{\varepsilon }^{\left(1\right)}+v_{\varepsilon }^{\left(2\right)}\right]},$$ (3)

where V represents the volume of the Schwarz P structure and $v_{\varepsilon }=\frac{w}{V}$ represents the strain energy density.

We intend to reduce the elastic modulus of the Schwarz P bone scaffold, and the optimization framework is expressed in Equation 4 (Jiang et al., 2024).

$$\left\{\begin{array}{l} \text{find}v_{\varepsilon }=\left(v_{\varepsilon }^{\left(1\right)},v_{\varepsilon }^{\left(2\right)}\right)\\ minf\left(v_{\varepsilon }\right)=\frac{18v_{\varepsilon }^{\left(1\right)}v_{\varepsilon }^{\left(2\right)}-2{\left[v_{\varepsilon }^{\left(2\right)}\right]}^{2}}{3\left[3v_{\varepsilon }^{\left(1\right)}+v_{\varepsilon }^{\left(2\right)}\right]},\\ \text{subject to}\left\{\begin{array}{l} 0.5\leq V_{\text{frac}}\leq 0.8\\ t\geq 0.2\text{mm} \end{array}\right. \end{array}\right.$$ (4)

where $V_{\text{frac}}$ represents the volume fraction of the Schwarz P scaffold and t represents the thickness of the scaffold.

**FIGURE 1:**
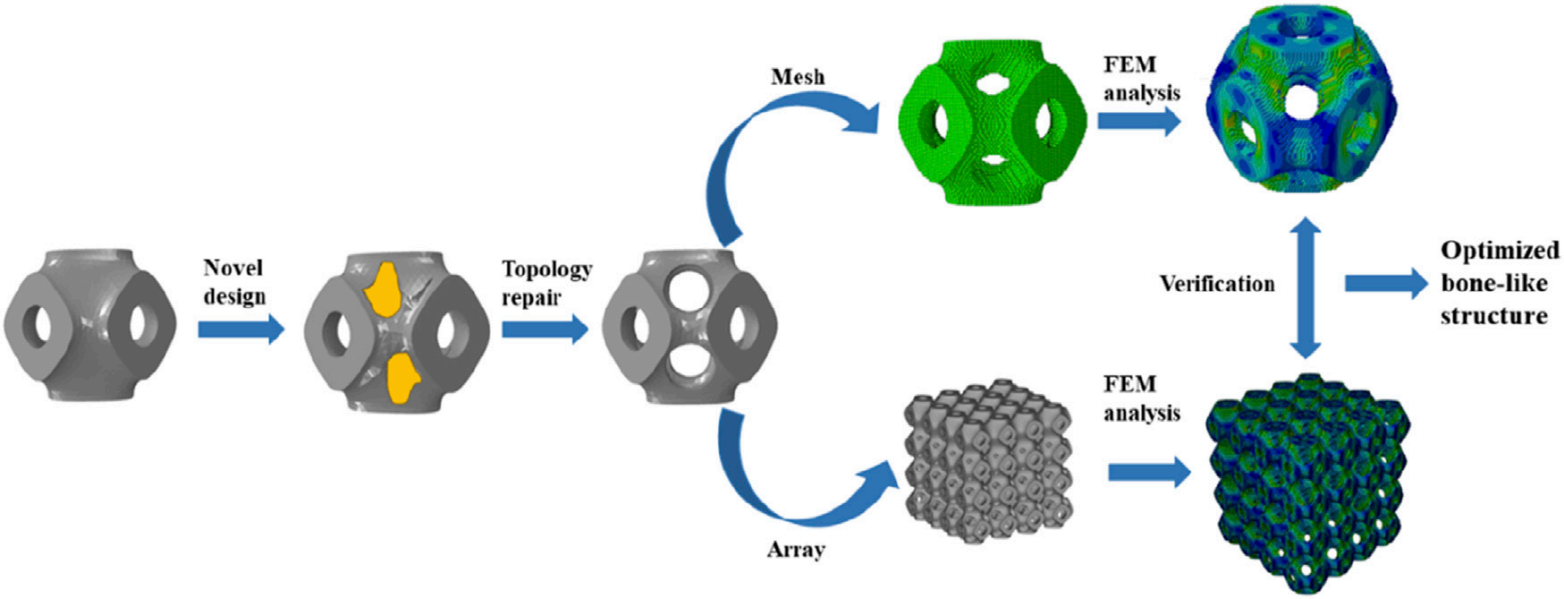
Schematic diagram of the design process of the novel structure.

**FIGURE 2:**
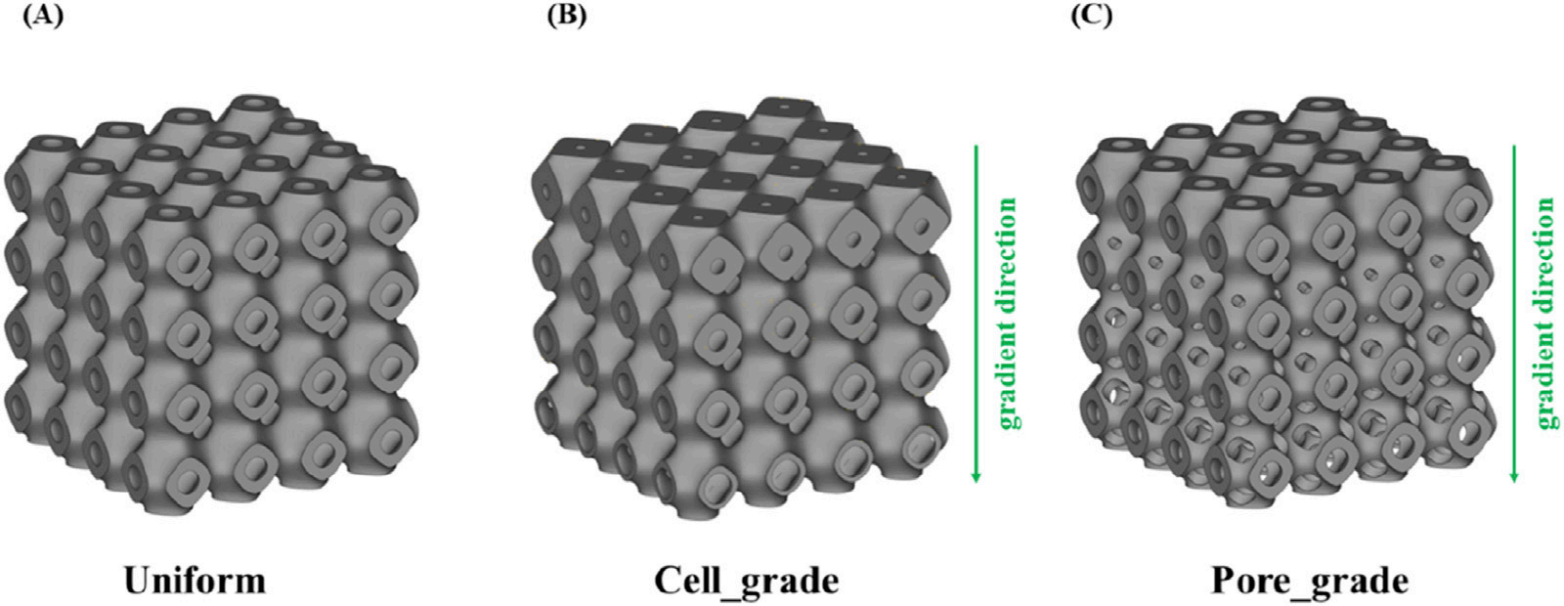
Three types of bone scaffolds. **(A)** Uniform Schwarz P bone scaffold with a porosity of 65%. **(B)** Cell gradient Schwarz P bone scaffold with a porosity of 65%. **(C)** Multi-functional pore-graded Schwarz P bone scaffold with a porosity of 65%.

Calculations were carried out using Abaqus (v2023, Dassault Systems SIMULIA Ltd. Providence, RI, United States) according to the optimization framework, and the Solid Isotropic Material Penalty (SIMP, one type of topology optimization) was used. As shown in Figure 1, the new structure was obtained by iterative computation, and then topological repair was performed to generate a unit cell with multi-functional pores, and a novel bone scaffold was designed based on the unit cell. According to the unit cell design method for multi-functional pores with different porosities, a unit cell graded Schwarz P bone scaffold with a porosity of 65% was obtained, as shown in Figure 2B. A linear transition was taken (Karuna et al., 2022), and the gradient transition method in this study are expressed as Equation 5.

$$\frac{V_{1}}{V_{2}}=2.0,$$ (5)

where $V_{1}$ and $V_{2}$ denote the volume sizes of the top unit cell and the bottom unit cell, respectively.

The strategy of the proposed optimized design was to add graded multi-functional pores, which can form a new topology of TPMS. The graded multi-functional pore bone scaffolds with a porosity of 65% were designed, as shown in Figure 2C. Two bone scaffolds with different gradient transitions were obtained with an overall porosity of 65%. It is worth noting that the structures with the same porosity imply the same volume of the solid material according to the definition of porosity, which can ensure comparability among different structures.

As shown in Figure 2A, a uniform Schwarz P bone scaffold with a porosity of 65% was also established as a control group. Therefore, three bone scaffolds with an overall porosity of 65% were compared to evaluate their performances. Similarly, two sets of bone scaffolds with porosities of 70% and 75% were designed, and the performance of these bone scaffolds was also compared using the same methods. All bone scaffold structures were generated using the software Flatt Pack (v3.31, University of Nottingham, United Kingdom), and the models were reprocessed using Materialise Magics (v24.0, Lovaine, Belgium). Additionally, the direction of gradient transition was set to the *Y*-direction for all graded bone scaffolds, while the *X* and *Z* directions remained unchanged.

### 2.2 Mechanical simulations and analysis

To evaluate the mechanical behavior of the novel scaffolds, the FE simulation was performed. Based on the parameters of the stiffness matrix, the Zener anisotropy indexes were calculated. The elastic moduli and Zener anisotropy indexes were used to evaluate the performance of novel scaffolds. For the FE simulations, the boundary conditions were set as Equations 6, 7:

$$\left\{\begin{array}{l} \Delta l_{x}{|}_{x=l_{x}}=0.001l_{x}\\ \Delta l_{x}{|}_{x=0}=\Delta l_{y}{|}_{y=0}=\Delta l_{y}{|}_{y=l_{y}}=\Delta l_{z}{|}_{z=0}=\Delta l_{z}{|}_{z=l_{z}}=0 \end{array}\right.,$$ (6)

$$\left\{\begin{array}{l} \Delta l_{x}{|}_{z=l_{x}}=0.0005l_{z},\Delta l_{z}{|}_{x=l_{z}}=0.0005l_{x}\\ \Delta l_{z}{|}_{x=0}=\Delta l_{y}{|}_{y=0}=\Delta l_{y}{|}_{y=l_{y}}=\Delta l_{z}{|}_{z=l_{z}}=\Delta l_{x}{|}_{z=0}=0. \end{array}\right.$$ (7)

The material of the bone scaffold was set as TC4 (i.e., a titanium alloy material) with a Young’s modulus of 110.0 GPa and a Poisson’s ratio of 0.34, which is widely used in bone implants (Montazerian et al., 2017). The parameters in Equation 2 were obtained by calculating the effective elastic modulus of the bone scaffold (Jiang et al., 2024). The elastic constants can be calculated as follows (Peng et al., 2023).

$$C_{ij}=\bar{\sigma }=\frac{1}{V}\int_{V}\;\sigma_{ij}\text{dV}.$$ (8)

Regarding the convergence analysis of the mesh, an FE model with an element size of 0.06 mm was used in this study, and results were independent of the mesh size (Jiang et al., 2024). To evaluate the mechanical anisotropy of the bone scaffold, the Zener anisotropy index, which is most commonly used to evaluate the anisotropic properties of materials, was used, and its expression is given as below (Chen et al., 2019).

$$A=\frac{2C_{44}}{C_{11}-C_{12}}.$$ (9)

When the Zener anisotropy index is equal to 1, the structure is isotropic, and when larger than 1, the anisotropy is more pronounced. After obtaining all the elastic constants $C_{ij}$ through Equation 8, the Zener anisotropy index for each structure can therefore be calculated by substituting the elastic constants into Equation 9. Additionally, the stiffness matrix of bone scaffolds was homogenized using MATLAB (R2023a, MathWorks, Inc., Natick, Massachusetts, United States), and each Young’s modulus surface was colored according to the magnitude of the effective stiffness.

### 2.3 Mass transport simulations and analysis

**FIGURE 3:**
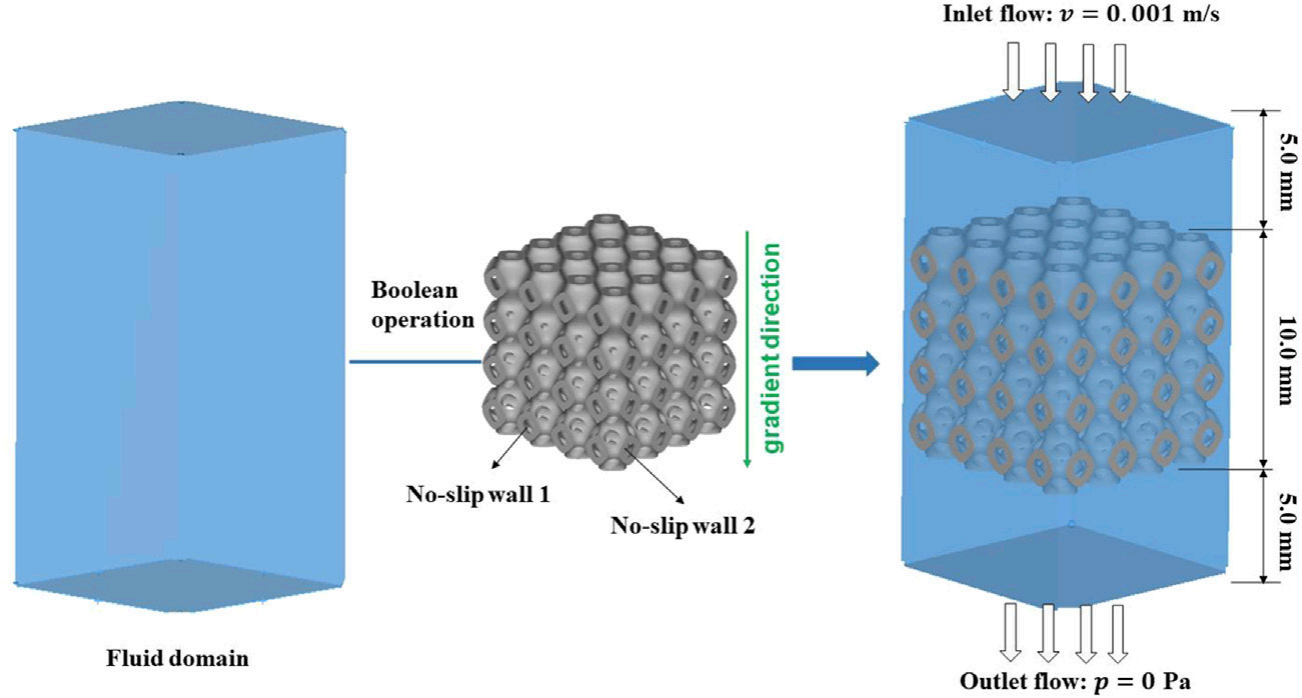
Modeling process of the fluid domain and the boundary conditions in CFD analysis.

The mass transport capacity of bone scaffolds was mainly evaluated by the permeability, and high permeability can facilitate nutrient transport and accelerate bone growth. Therefore, CFD was performed in COMSOL (v6.0, COMSOL Multiphysics, Stockholm, Sweden) to simulate the fluid flow process in the bone scaffolds. The permeability of the bone scaffolds was calculated before and after the optimal design of the structure. The unit cells were arrayed into a 4 × 4 × 4 bone scaffold structure with an overall size of 10.0 mm × 10.0 mm × 10.0 mm. Since the analysis of permeability needs to be carried out on the fluid domain, Boolean operations were performed on the bone scaffold to obtain the fluid region. To avoid the boundary conditions caused by the inlet and outlet regions, a fluid domain of 10.0 mm × 10.0 mm × 5.0 mm was established at both the fluid inlet and outlet, which can ensure that a more stable state was achieved in the fluid through the region of the bone scaffold. In this way, a 10.0 mm × 10.0 mm × 20.0 mm parallel hexagonal fluid domain was established, as shown in Figure 3. The specific boundary conditions for permeability calculations were set in TPMS bone scaffolds (Zhang et al., 2020). The rate of inlet flow was 0.001 m/s, and the outlet pressure was 0.0 Pa. The inner and outer surfaces of the bone scaffolds were set to no-slip walls. The permeability was based on Darcy's law expressed as Equation 10 (Peng et al., 2023).

$$Re=\frac{v\rho D}{\mu },$$ (10)

where Re denotes the Reynolds number; v denotes the fluid flow rate (m/s); ρ denotes the fluid density (kg/m^3^); and D denotes the radius of the pore. The pressure drop between the inlet and outlet can be obtained by CFD calculation, and the permeability K of the bone scaffold can be calculated as Equations 11, 12.

$$K=\frac{v\mu L}{\Delta P},$$ (11)

$$v=\frac{Q}{A},$$ (12)

where K denotes the permeability of the bone scaffold; μ denotes the coefficient of kinetic viscosity of the fluid (Pa ⋅ s); L denotes the straight length in the fluid direction (m); ΔP denotes the pressure difference between the inlet and outlet (Pa); Q denotes the volume of fluid flowing through the structure per unit of time ( $m^{3}/s$ ); and A denotes the cross-sectional area of the fluid domain ( $m^{2}$ ). The fluid flowing through the bone scaffold was set to be water with the following specific parameters: ρ= 1000 kg/ $m^{3}$ ; μ=0.001 Pa ⋅ s; v=0.001 m/s. The fluid volume of the bone scaffold was set to be water.

## 3 Results

### 3.1 Mechanical properties of the novel structures

#### 3.1.1 Comparison of the effective elastic modulus among three types of bone scaffolds

**FIGURE 4:**
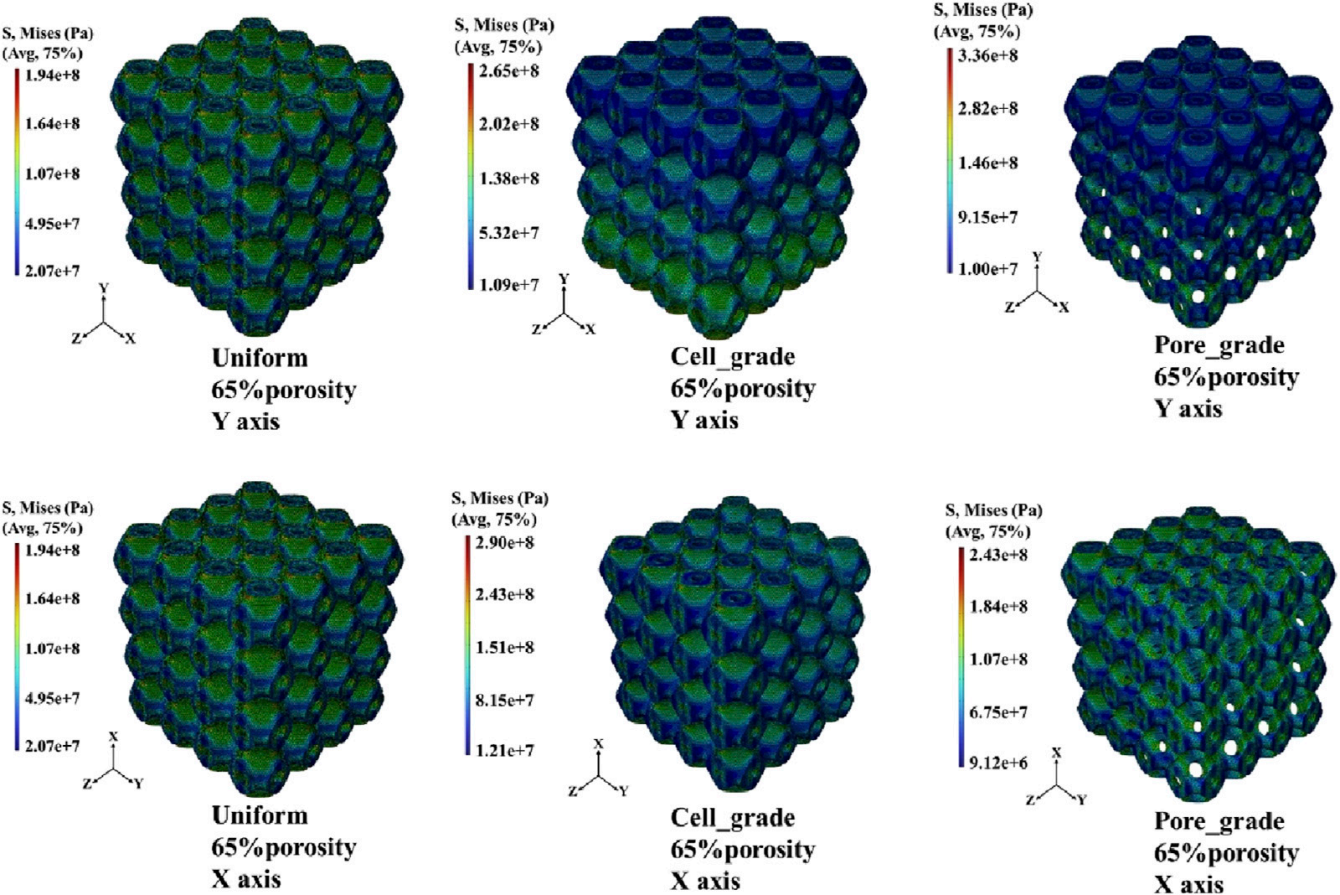
Von Mises stress distribution of three types of Schwarz P bone scaffolds with porosity 65% under uniaxial compression.

**FIGURE 5:**
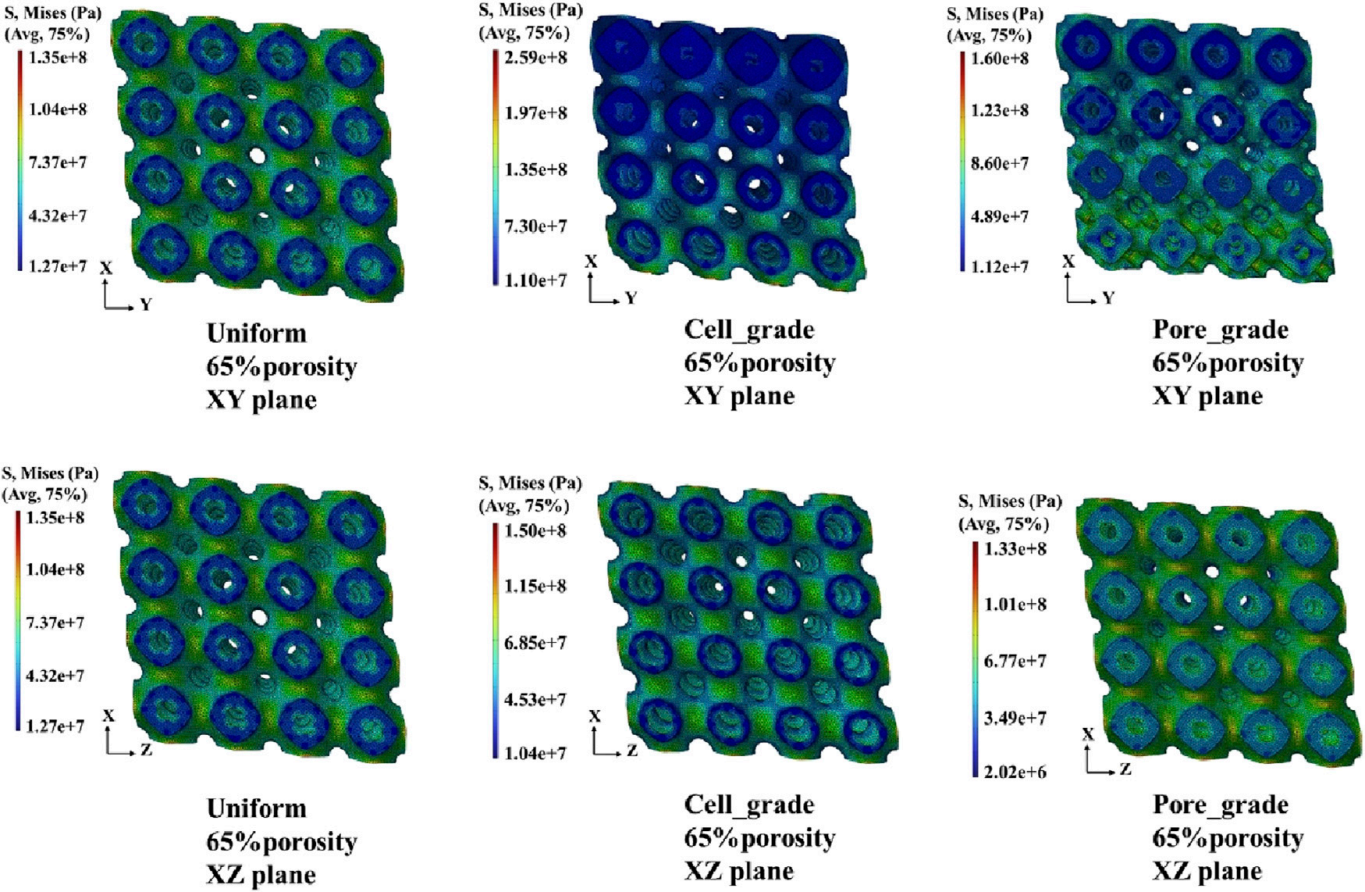
Von Mises stress distribution of three types of Schwarz P bone scaffolds with porosity 65% under unidirectional shear loading.

The von Mises stress distribution of the three bone scaffolds was calculated. The von Mises stress distribution under uniaxial compression for the scaffolds at 65% porosity is presented in Figure 4. The uniform Schwarz P scaffold exhibited a homogeneous and periodic stress distribution, which aligns with the typical properties of TPMS structures. In contrast, for the multi-functional pore-graded Schwarz P scaffolds, a more pronounced variation in stress distribution was observed in the *Y*-direction due to the gradient transition from the top to the bottom. A smoother stress variation was found in the multi-functional pore-graded scaffold compared to the single-cell gradient scaffold, indicating improved homogeneity in stress distribution and reduced directional variability in mechanical properties. Similar patterns were observed in the unidirectional shear von Mises stress distribution (Figure 5), where a gentler transition was noted in the multi-functional pore-graded scaffold compared to the single-cell gradient scaffold, while the non-gradient transition direction exhibited uniformity.

**FIGURE 6:**
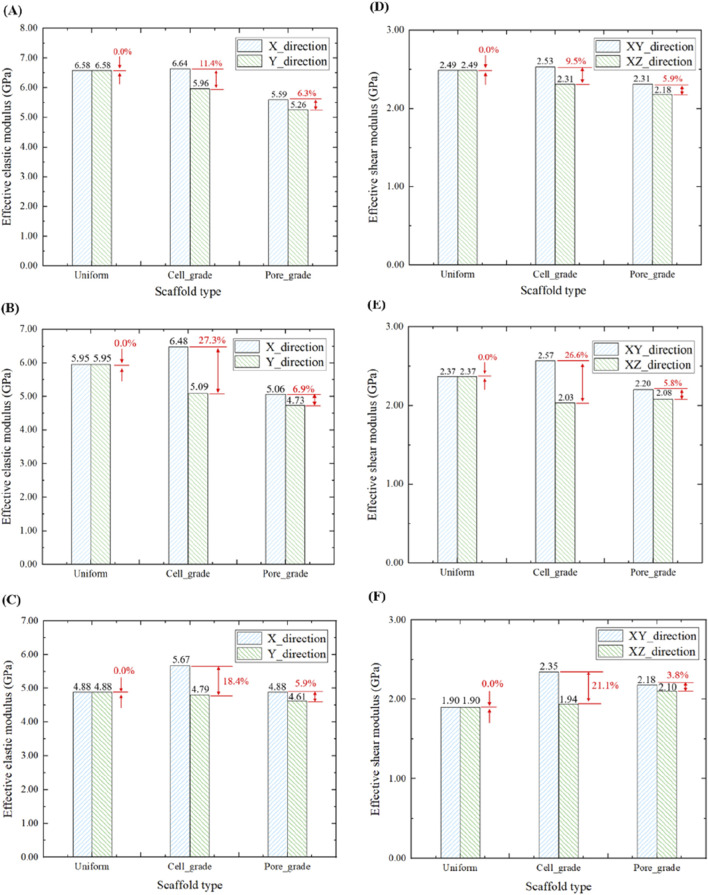
Comparison of the elastic moduli in three groups of bone scaffolds. **(A)** Effective elastic modulus of bone scaffolds with a porosity of 65%. **(B)** Effective elastic modulus of bone scaffolds with a porosity of 70%. **(C)** Effective elastic modulus of bone scaffolds with a porosity of 75%. **(D)** Effective shear modulus of bone scaffolds with a porosity of 65%. **(E)** Effective shear modulus of bone scaffolds with a porosity of 70%. **(F)** Effective shear modulus of bone scaffolds with a porosity of 75%.

Comparison in the effective elastic moduli in the three groups of bone scaffolds at the porosities of 65%, 70%, and 75% is illustrated in Figure 6. The uniform bone scaffold was served as a control in the comparison. For the unit cell graded bone scaffolds with the porosities of 65%, 70%, and 75%, the difference in the effective compressive moduli between the *X* and *Y* directions was found to be 11.4%, 27.3%, and 18.4%, respectively, while the corresponding values for the bone scaffolds with functionally graded pores were 6.3%, 6.9%, and 5.9%, respectively. Therefore, it can be concluded that the difference in the effective compressive modulus between *X* and *Y* directions in the bone scaffolds with functionally graded pores was significantly reduced at different porosities.

By analyzing the elastic modulus of bone scaffolds on the three types, several results were obtained. At a porosity of 65%, the effective compressive modulus of the uniform bone scaffold was 6.58 GPa in both *X* and *Y* directions. For the unit cell graded bone scaffold, the elastic modulus was 6.64 GPa in the *X* direction and 5.96 GPa in the *Y* direction. The bone scaffold with multi-functionally graded pores exhibited a compressive modulus of 5.59 GPa in the *X* direction and a compressive modulus of 5.26 GPa in the *Y* direction. At a porosity of 70%, the uniform bone scaffold had an effective compressive modulus of 5.95 GPa in both *X* and *Y* directions. The unit cell gradient bone scaffold exhibited an elastic modulus of 6.48 GPa in the *X*-direction and an elastic modulus of 5.09 GPa in the *Y*-direction. The bone scaffold with multi-functionally graded pores displayed an elastic modulus of 5.06 GPa in the *X*-direction and an elastic modulus of 4.73 GPa in the *Y*-direction. At a porosity of 75%, the uniform bone scaffold had an elastic modulus of 4.88 GPa in both *X* and *Y* directions. The unit cell gradient bone scaffold showed an elastic modulus of 5.67 GPa in the *X*-direction and an elastic modulus of 4.79 GPa in the *Y*-direction, while the bone scaffold with multi-functionally graded pores exhibited elastic moduli of 4.88 GPa and 4.61 GPa in the *X* and *Y* directions, respectively. It was shown that the unit cell graded bone scaffolds had a larger effective compressive modulus in the non-gradient transition direction when the porosity was the same, which may exacerbate the occurrence of stress shielding and was not conducive to the long-term stability of the bone scaffolds. In contrast, bone scaffolds with multi-functionally graded pores exhibited smaller effective compressive moduli in both gradient transition and non-gradient transition directions, which is beneficial for mitigating stress shielding. For the effective shear modulus, at three different porosities of 65%, 70%, and 75%, the difference in the effective shear modulus of the unit cell graded bone scaffolds in different directions was 9.5%, 26.6%, and 21.1%, respectively, while those in the bone scaffolds with multi-functionally graded pores were 5.9%, 5.8%, and 3.8%, respectively. Similar to the analysis of effective compressive modulus, the difference in the effective shear modulus of the bone scaffolds with multi-functionally graded pores in different directions reduced greatly at different porosities.

#### 3.1.2 Comparison of the spatial distribution of effective elastic modulus among three types of bone scaffolds

**FIGURE 7:**
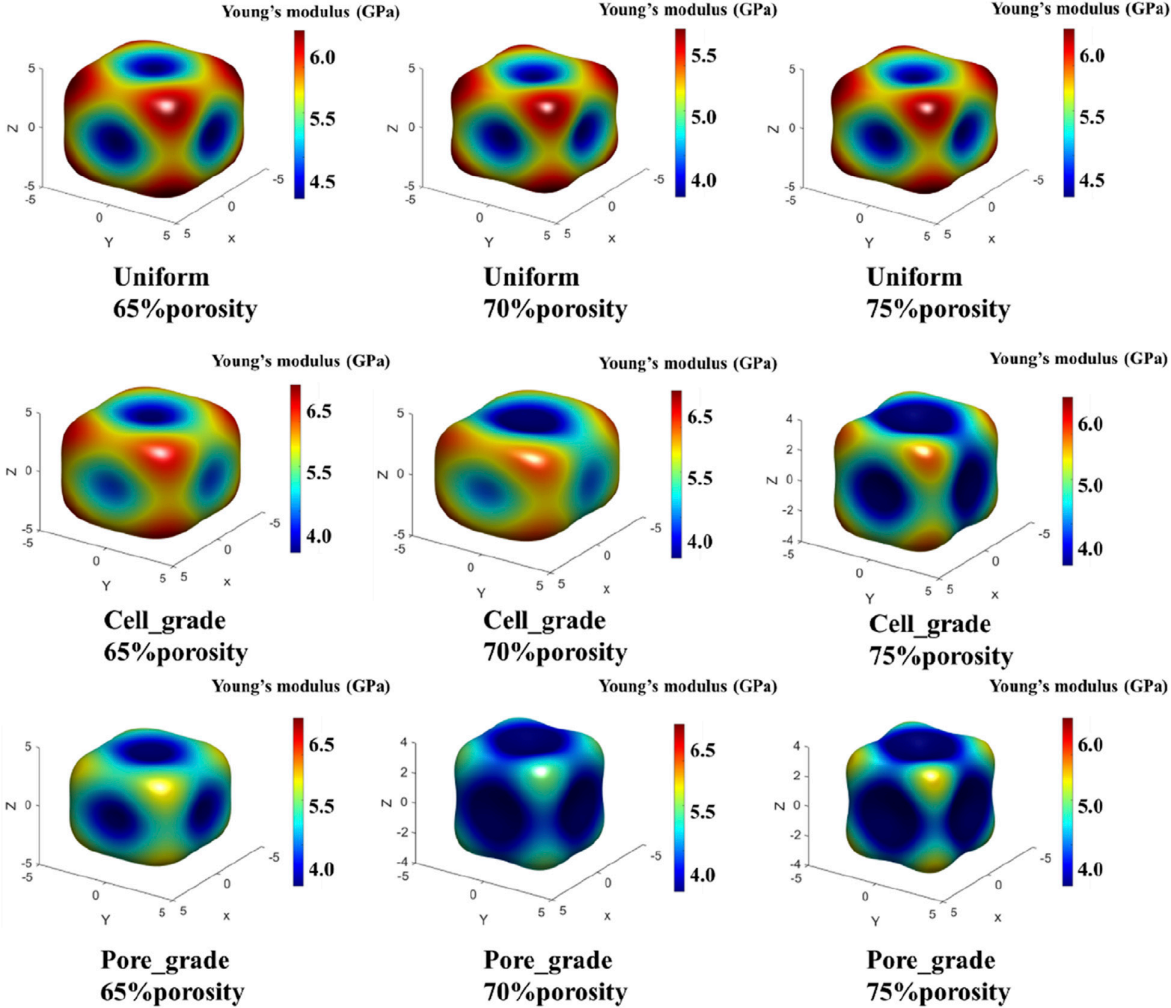
Comparison of the three-dimensional spatial distribution of the effective elastic modulus for three types of Schwarz P bone scaffolds.

The anisotropy of bone scaffolds was analyzed using the spatial distribution of the elastic modulus in three dimensions, as shown in Figure 7. Since the Schwarz P structure is cubic symmetric, there are only three independent elastic constants *C*_11_, *C*_12_, and *C*_44_ in the stiffness matrix. However, a porosity gradient is introduced to the functionally graded Schwarz P bone scaffold in one direction, so it is no longer a cubically symmetric structure, and the independent elastic constants are increased by 2. The Zener anisotropy index in Equation 9 was calculated using the three independent elastic constants *C*_11_, *C*_12_, and *C*_44_, which was not suitable for the functionally graded bone scaffolds. Thus, the anisotropy of the bone scaffolds was examined through the three-dimensional spatial distribution of the elastic modulus.

The distribution of elastic modulus for both the unit cell graded bone scaffolds and the bone scaffolds with multi-functionally graded pores was similar. The elastic modulus was lower in the central region of the eight faces of the cube and higher in the direction of the eight corner points of the cube. At all three porosities with 65%, 70%, and 75%, the difference between the highest and the lowest elastic modulus of the bone scaffolds with multi-functionally graded pores was relatively smaller, whereas that of the unit cell gradient bone scaffold was relatively larger. For instance, at a porosity of 65%, although both scaffolds showed the highest elastic modulus in the direction of the cube corner point, the difference among the moduli in the direction of the cube corner point and the other directions was larger in the unit cell graded bone scaffold, implying greater anisotropy. In contrast, the anisotropy of bone scaffolds with multi-functionally graded pores was not as significant as that of the unit cell gradient bone scaffold.

### 3.2 Comparison in mass transport capacity between functionally graded Schwarz P bone scaffolds

**FIGURE 8:**
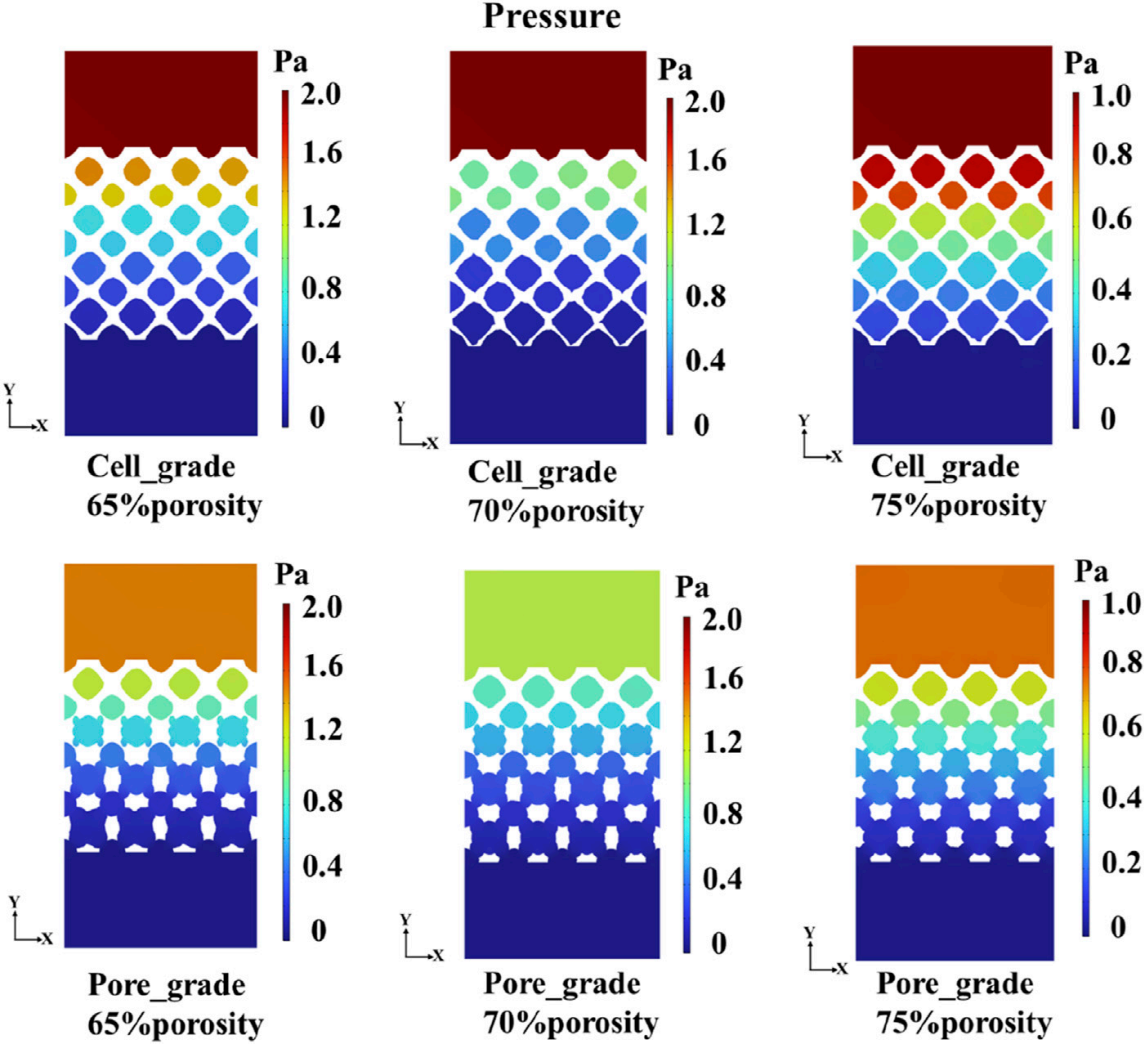
Pressure distribution of functionally graded Schwarz P bone scaffolds.

Comparisons in the mass transport capacity between two functionally graded bone scaffolds were conducted. Jiang et al. (2024) investigated the significantly higher mass transport capacity of unit cell gradient bone scaffolds compared to uniform bone scaffolds. Accordingly, the mass transport capacity of two functionally graded bone scaffolds was only compared in the study. A cross-section through the center of the multi-functional pore was chosen to represent the pressure drop in the bone scaffold structure, as shown in Figure 8. For each porosity, the pressure drop of the graded multi-functional pore bone scaffold was smaller than that of the unit cell gradient bone scaffold, and the reduction in pressure drop is favorable to the enhancement in permeability. Therefore, better mass transport capacity was achieved by adding the graded multi-functional pores. Additionally, from the fluid flow domain analysis, it can be seen that the fluid flow domain of both gradient bone scaffolds gradually increased along the gradient direction. This was because the porosity was gradually increasing from the top to the bottom of the gradient bone scaffold, and the corresponding fluid flow domain was getting bigger, which may be favorable for mass transport.

**FIGURE 9:**
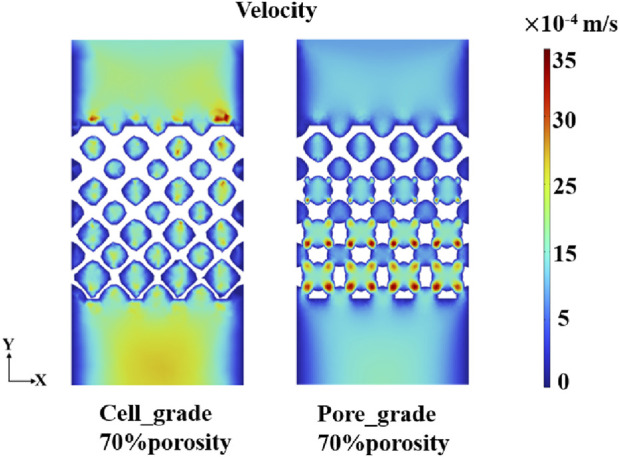
Fluid velocity distribution of unit cell gradient bone scaffolds with a porosity of 70% and graded multi-functional pore bone scaffolds with a porosity of 70%.

The flow region of the unit cell graded bone scaffolds was still independent on each other, despite the increase in the size of the region. In contrast, the flow region in the bone scaffolds with multi-functionally graded pores not only increased gradually but also achieved full connectivity near the bottom. The mass transport capacity can be greatly improved, due to the superiority of multi-functional pores. At a porosity of 70%, unit cell gradient bone scaffolds and bone scaffolds with multi-functionally graded pores were selected and analyzed for the velocity of fluid flow, as shown in Figure 9. It can be seen that the flow velocity in the bone scaffolds with multi-functionally graded pores increased with an increase in the fluid flow region, and the effect of flow velocity increase became more obvious as the flow region gradually connected.

**FIGURE 10:**
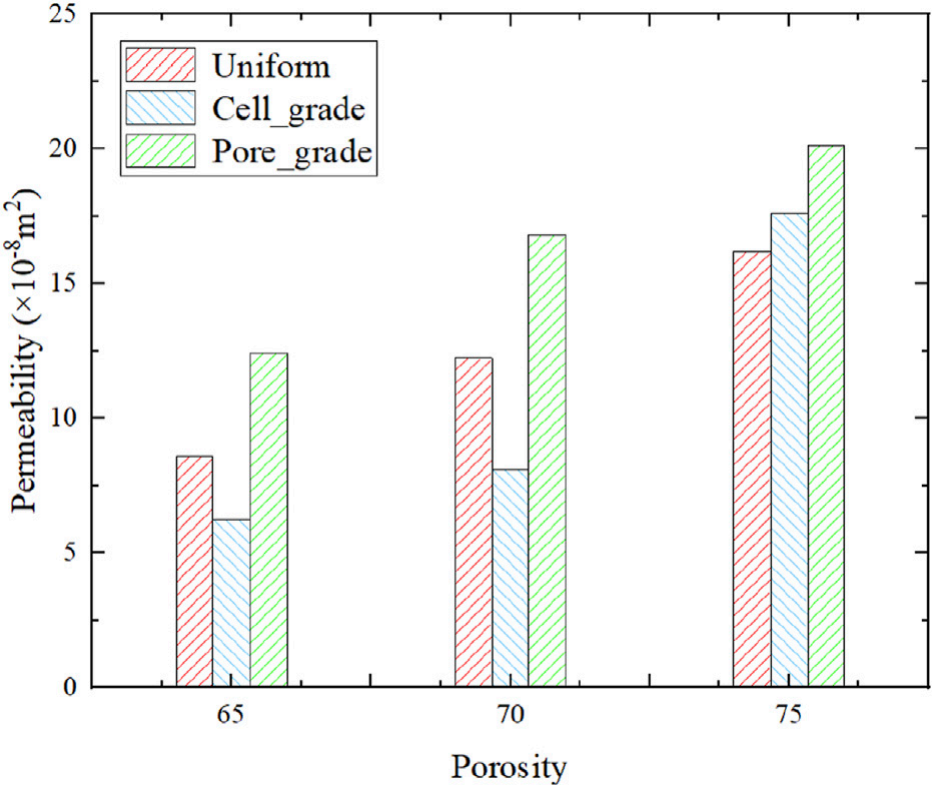
Permeability of functionally graded bone scaffolds before and after optimal design.

After obtaining the pressure drop of the bone scaffolds, the permeability can be calculated from Equation 11, as shown in Figure 10. The bone scaffolds with multi-functionally graded pores had the highest permeability at each porosity. The permeability of each bone scaffolds increased with the increase in porosity.

## 4 Discussion

Currently, bone scaffolds based on TPMS structures are widely used because their properties are closest to those of human bones. However, the performance of the TPMS bone scaffolds, such as elastic modulus, anisotropy, and permeability still cannot fully meet the needs of human bones. To address the problems, a novel bone scaffold with multi-functionally graded pores was proposed based on the TPMS structure. The performance of the designed bone scaffold was characterized and analyzed by numerical calculation methods. Some interesting findings were revealed in this study.

First, the elastic modulus of the novel bone scaffold was significantly lower, which can mitigate the effect of stress shielding. The optimized bone scaffolds with multi-functionally graded pores have a smaller difference on the effective compressive modulus and the effective shear modulus in different directions at three different porosities, namely, 65%, 70%, and 75%, which can meet the needs of some specific bone implants. The elastic modulus of the novel scaffold designed in the present study is closer to that of human bones, compared to the traditional scaffolds. It is reported in the literature that the elastic modulus of human cortex bones is approximately from 1.08 to 3.47 GPa (Sevilla et al., 2007; Wu et al., 2018), the elastic modulus of traditional scaffolds is approximately from 5.01 to 6.58 GPa (Khaleghi et al., 2021; Jiang et al., 2024; Li et al., 2024), and the elastic modulus of the scaffold in this study can reach approximately 4.61 GPa. Therefore, it is believed the optimized scaffold could better reduce the stress shielding, compared to traditional scaffolds, but this requires further direct investigation in the future. Moreover, the gradient transition structure of the novel bone scaffold was similar to the gradient transition structure of human natural bone, i.e., geometrically similar to human bone.

Second, the differences between the highest and lowest moduli of elasticity for bone scaffolds with multi-functionally graded pores at porosities of 65%, 70%, and 75% were relatively small, whereas the differences were relatively large for bone scaffolds with unit cell gradients. This also confirms that the elastic modulus of the bone scaffolds with multi-functionally graded pores matches better with that of human bone.

Third, the mass transport capacity of designed bone scaffolds with multi-functionally graded pores was higher than that of unit cell gradient bone scaffold and uniform bone scaffold, which can be beneficial for nutrient transport and cell growth. It should be noted that the permeability of the bone scaffold may also be anisotropic due to the gradient variation in the *Y* direction. However, the anisotropic analysis of permeability is not the focus of the study. The permeability of bone scaffolds was analyzed along the gradient transition direction to obtain the fluid flow velocity and structural permeability. The permeability of designed bone scaffolds with multi-functionally graded pores was higher than that of the unit cell gradient bone scaffolds, which may be greater mass transport capability.

Some limitations in the present work should be noted. First, the experimental test of the novel bone scaffold was not performed. The experimental part has been demonstrated by the uniform bone scaffolds and the unit cell gradient bone scaffolds (Peng et al., 2023). Therefore, it can be assumed that the results in this study were reliable. Additionally, it should be noted that the calculations were performed using linear-elastic material settings, without considering the plastic phase. This aspect will be addressed in future research by defining a complete material constitutive model for more accurate calculations, and the compressive and tensile strength could be evaluated afterward. Second, the mass transfer capability was investigated only in one direction as the direction of the gradient transition is a very important point to focus on. Third, the fluid used in the mass transport simulations was simplified to Newtonian water. It is acknowledged that the assumption of using Newtonian water in these simulations oversimplifies the rheological behavior of bone tissue fluids (e.g., blood and bone marrow). We thus incorporate a non-Newtonian fluid model in future work for a more accurate assessment (Wang et al., 2022). Nevertheless, the use of water as the fluid is considered reliable for studies focusing on the overall feasibility of mass transport. Last but not the least, cell and animal experiments have not been conducted either. This will be studied in depth in the future in order to further realize clinical applications.

## 5 Conclusion

In this study, a novel functionally graded TPMS bone scaffold was designed by introducing multi-functional pores as a novel geometric variable into traditional bone scaffolds. The performance of the novel new bone scaffolds was evaluated and compared to those of uniform and unit cell graded scaffolds. The main findings are as follows:

1) Compared to the commonly used unit cell graded bone scaffolds, the effective compression modulus and effective shear modulus of the bone scaffolds with multi-functionally graded pores are significantly lower, which contributes to the reduction in the stress-shielding effect.
2) Compared to the traditionally cell graded bone scaffolds, the elastic modulus of the bone scaffolds with multi-functionally graded pores was more spatially and uniformly distributed, with smaller differences in the values of the elastic modulus in different directions, which aligns more closely with the goal of mimicking the mechanical behavior of natural bone.
3) The designed bone scaffolds with multi-functionally graded pores have a higher permeability compared to the unit cell graded bone scaffolds and uniform bone scaffolds, suggesting greater mass transport capabilities.

This study focuses on designing a novel Schwarz P structure with multi-functional pores by using a unit cell optimization method. These pores were used to create graded bone scaffolds with functional gradients and mechanical anisotropy, suitable for implantation in areas like the femur and vertebrae. The results demonstrate that, compared to the widely used bone scaffolds, the bone scaffolds with multi-functionally graded pores exhibit lower directional variation in effective modulus and possess higher mass transport capabilities. The smaller directional difference in effective modulus results in lower stress shielding, whereas the enhanced mass transport capacity promotes nutrient transport and supports cell growth. The novel graded multi-functional pore scaffolds successfully met the mechanical anisotropy and mass transport requirements needed for bone scaffolds with excellent performance. The novel TPMS bone scaffold offers two key advantages: 1) its gradient porosity transition structure closely resembles the natural gradient structure found in human bone, providing a geometric similarity that is beneficial for bone integration, and 2) the equivalent compressive modulus and shear modulus exhibit minimal directional variation, which results in a more isotropic mechanical performance. This feature is particularly important for implantation in load-bearing regions such as the vertebrae, where mechanical homogeneity is essential to ensure proper function and support. In the end, this design approach expands the design space of functional gradient TPMS bone scaffolds and provides a theoretical basis for the development of high-performance bone scaffolds.

## Data Availability

The raw data supporting the conclusions of this article will be made available by the authors, without undue reservation.
